# Agenesis of the corpus callosum: a rare association with Ehlers-Danlos syndrome

**Published:** 2020

**Authors:** Motahareh AFRAKHTEH, Mostafa ALMASI-DOOGHAEE, Fahimeh HAJI AKHOUNDI

**Affiliations:** 1 Department of Neurology, Firoozgar Hospital, Iran University of Medical Sciences, Tehran, Iran

**Keywords:** Ehlers-Danlos syndrome, spondylolisthesis, Agenesis of the corpus callosum.Introduction

## Abstract

Ehlers-Danlos syndrome (EDS) is a rare congenital disorder of connective tissues which involves the skin and musculoskeletal system. There are also some reports for the involvement of the central and peripheral nervous systems. We want to present a very rare coassociation of EDS, spondylolisthesis, and Agenesis of the corpus callosum in an Iranian lady.

## Introduction

Ehlers-Danlos syndrome (EDS) is a rare congenital disorder of connective tissues, which is mainly demonstrated through skin hyper-elasticity, joint hyper-mobility, and internal organ abnormality, which is caused by a disorder in type II, III, and V collagens. Considering the affected collagen and biological and molecular pattern, the EDS has various kinds. The most common of which is Classical EDS. Also, depending on the type of EDS, the patterns of inheritance pedigree are different, including autosomal recessive, autosomal dominant, or X-linked. The incidence of EDS is estimated to be 1 per 5000 to 1 per 100000 live births in different societies.  

Both central and peripheral nervous systems can be involved primarily or secondarily at the course of EDS. The most common central nervous system involvement accompanied by EDS is brain heterotopias. There is also limited evidence about the association between Agenesis of the corpus callosum (ACC) and EDS. The current research is an attempt to shed light on a case of EDS accompanied by ACC, which is a very rare association.

## Case Presentation

**Figure 1:**
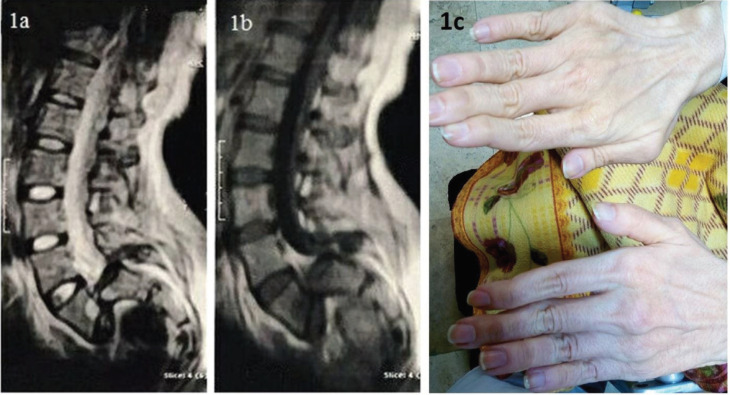
Sagittal T2-weighted (1a) and T1-weighted (1b) MRI of the lumbosacral spine revealed severe spondylolisthesis of L5-S1 vertebrae. The patient had thin and deformed fingers (1c).

**Figure 2:**
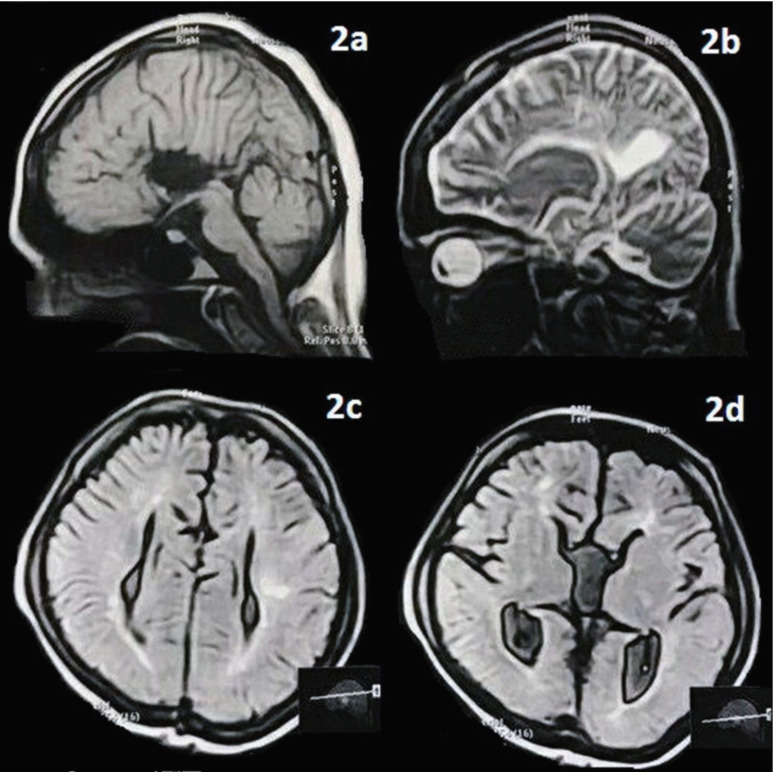
Sagittal T1-weighted (2a) and T2-weighted (2b) MRI of the brain revealed complete absence of corpus callosum and cingulate gyrus and radial arrangement of sulci. Axial Fluid-attenuated inversion recovery (FLAIR) MRI of the brain showed parallel lateral ventricles (2c), “race-car” appearance of lateral ventricles, and colpocephaly (2d). All of these findings suggest Agenesis of the corpus callosum (ACC).

The case we studied was a 22 years old female patient who was diagnosed with EDS type VI based on the clinical findings, including soft, slightly hyper-extensible skin, skin fragility, kyphoscoliosis, and increased range of motion of joints. The patient was admitted to the orthopedic ward for repair surgery of severe disabling spondylolisthesis (Figures 1a and 1b). She had a history of fever accompanied by seizures at the age of six. Preliminary examinations indicated suffering from long, thin, and deformed fingers (Figure 1c) as well as hyperlaxity of ligaments. Further neurologic examinations revealed paraparesis (motor force of 2/5), bilateral action and intention tremor, right side Babinski sign, and bilateral Huffman sign. The deep tendon reflexes (DTR) of upper limbs were 3+ (exaggerated) on the right side and 2+ (normal) on the left side. Also, the knee and ankle reflexes on the right side were slowed down to 1+, but reflexes of knee and ankle on the right side were missing. Besides, senses of touch and pain in the lower limbs were completely impaired, but no sensory level was found at the examination of the trunk. For this patient, spondylolisthesis gives possible grounds for flaccid paraparesis and sensory symptoms; however, Babinski sign, Huffman sign, and exaggerated DTR could be attributed to another upper motor neuron lesion. To shed light on this issue, a brain MRI was conducted, which showed complete absence of corpus callosum and cingulate gyrus (Figures 2a and 2b), parallel lateral ventricles (Figure 2c), “race-car” appearance of lateral ventricles, and colpocephaly (Figure 2d); in favor of Agenesis of the corpus callosum (ACC).

## Discussion

Several studies have reported evidence on the EDS, one of which is structural anomalies in the brain and spinal cord. Periventricular heterotopia is one of these anomalies, which is accompanied by a mutation in the FLNA gene.  Single reports of other structural anomalies such as Agenesis of the corpus callosum, Polymicrogeria, dilatation of the fourth ventricle of the cerebrum, and supra-cerebral cistern and lateral ventricle a disproportional enlargement of the anterior horn of lateral ventricle are also recorded. Our patient was a case of ACC with EDS that similar cases are reported elsewhere. For example, Antonio Federico and colleagues (1997) reported a patient that had ACC with quadric-cuspid aortic valve4; however, our patient demonstrated ACC with spondylolisthesis, which is a structural anomaly of the spinal column, the possible mechanism of which is defined as severe laxity in ligaments.  Other structural anomalies related to the nervous system include meningeal cyst spine, Chiari type I malformation^,^, spondylolisthesis, and herniation of vertebral disc . 

Besides, there are other neurologic demonstrations about the course of EDS, including neuropathy, epilepsy, fatigue, developmental features, pain, and headache8, the pain similar to that of Migraine with and without aura, tension headache, tension-migraine headache complexity, and post-traumatic headache. These findings may occur due to the co-association of primary congenital anomalies of the nervous system with EDS or secondary involvement of the nervous system as a result of connective tissue abnormalities related to the EDS. Similarly, vascular changes can cause neurologic symptoms, particularly stroke, in the forms of intracranial aneurism, subarachnoid hemorrhage, arterial dissection, and cavernous sinus fistula. 

In summary, in the current study, we reported a rare case of ACC and spondylolisthesis in a patient with EDS type VI. Although these demonstrations are far from regular, physicians should be aware of these symptoms and, in the case of diagnosis, they have to refer the patient and other family members for further investigations.

## In Conclusion

Ehlers Donlos syndrome type VI is a connective tissue disorder and may be accompany with agenesis of the corpus callosum and spondylolisthesis.
